# Identification of mitogenic factor in rice bran for better mammalian cell culture

**DOI:** 10.1186/1753-6561-7-S6-P107

**Published:** 2013-12-04

**Authors:** Yoko Suzuki, Satoko Moriyama, Masayuki Taniguchi, Shigeru Moriyama, Takuo Tsuno, Satoshi Terada

**Affiliations:** 1 University of Fukui, Fukui, 910-8507, Japan; 2 Niigata University, Niigata, 950-2102, Japan; 3 Tsuno Food Industrial Co., Ltd, Katsuragi-cho, Wakayama, 649-7122, Japan

## Introduction

In cell culture for biopharmaceutical production, serum-free culture is required in order to avoid the risks associated with components of mammal origin such as BSE. Although many serum-free medium have been developed, there is yet room for improvement and protein hydrolysates from crops are widely used as additives to improve the culture.

We found that rice bran extract (RBE), not hydrolysate, successfully improved the proliferation of various cells as well as recombinant protein production of CHO cells when RBE was added into serum-free culture. Several studies have been done and reported that rice bran has antioxidant potential [1,2] and a rice bran 57-kDa protein showed cell adhesion activity for murine Lewis lung carcinoma cells [3].

RBE contains various components such as proteins and the factors activating mammalian cells are not identified yet. In this study, we aim to identify the effective factor in RBE.

Our colleague reports that heavier molecular weight fraction of RBE improves the proliferation of various cells. Additionally, protein is the most abundant component in RBE. Together with them, some of the proteins in RBE would be the effective factor or the mitogen. We first determined whether some of the proteins in RBE are the bio-active factor or not, and then tried to identify which protein in RBE is the bio-active factor.

## Materials and methods

### Effect of heat treatment on RBE

RBE was autoclaved at 121°C for 20 minutes. The heated RBE was supplemented into the culture of murine hybridoma cell line 2E3-O. Hybridoma cells were cultured in 24-well plate (Sumitomo Bakelite, Japan) with 1 ml ASF104 medium (Ajinomoto, Japan) in the presence of heated RBE. On day 3, viable cell number was determined by trypan blue dye exclusion with hemocytometer.

### Effect of trypsin treatment on RBE

RBE was digested with trypsin at 37°C for 24 hours. The treated RBE was SDS electrophoresed to confirm RBE was digested and to decide the condition. The trypsinized RBE was supplemented to the culture of hybridoma cells. On day three, viable cell number was determined.

### Proteins in Rice Bran

Two kinds of oryzacystatins are known in rice bran; oryzacystatin I and II. Antiserum against both oryzacystatin I and II was prepared, and mobilized in HiTrap Protein A column (GE Healthcare, USA). Using affinity chromatography, oryzacystatin I and II were eluted with 100 mM Glycine-HCL (pH 2.9) containing 2 M Urea. All purification steps were done at 4°C.

The purified oryzacystatin was supplemented to the culture of hybridoma cells. On day three, viable cell number was determined.

## Results and discussion

### Autoclaved RBE lost mitogenic activity

While un-heated RBE successfully improved the proliferation, autoclaved RBE failed, suggesting that mitogenic factors in RBE would be heat-sensitive.

### Trypsinized RBE lost mitogenic activity

**Figure 1:**
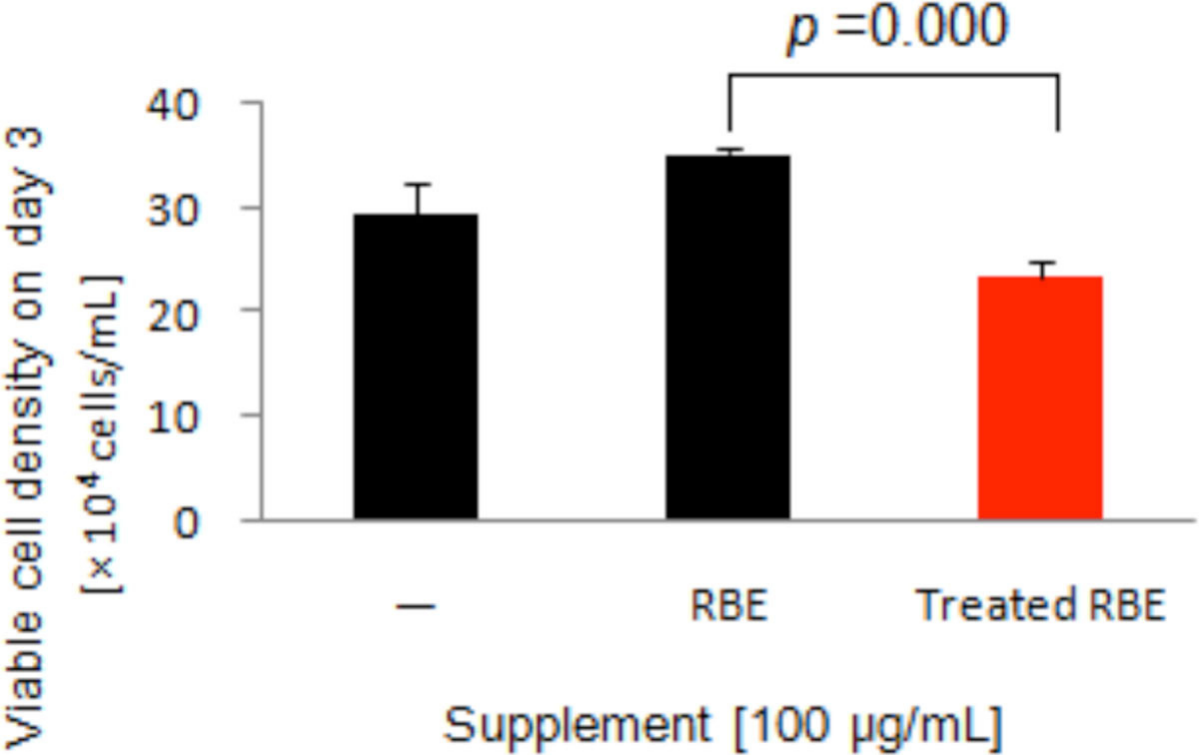
**Effect of trypsinized RBE on hybridoma cell growth**. RBE was digested with trypsin at 37°C for 24 hours.

Most of proteins including 31 kDa protein of RBE were successfully digested with trypsin. Although the proliferation of the cells treated with undigested RBE was stimulated, that of the cells treated with trypsinized RBE was not, suggesting that effective factors in RBE would be some proteins. Effect of trypsinized RBE on hybridoma cell growth is shown in Figure 1.

### Purified Oryzacistatin from RBE did not improve the cellular proliferation

Oryzacystatin obtained from RBE did not improve the culture of hybridoma, suggesting that oryzacystatin would not be mitogen. Other proteins in RBE would have mitogenic effects on mammalian cells.

## Conclusions

RBE improves the culture of various cells. Both of autoclaved and trypsinized RBE had lost the mitogenic effect, suggesting that bio-active factors in RBE would be heat-sensitive ingredients, probably proteins.

Among abundant proteins in rice bran, oryzacystatin was purified from RBE and supplemented into the culture, but it failed to improve the culture. Other proteins in RBE will be tested to identify bio-active factor in RBE.
